# Public Perceptions of Personal Data Spaces for Citizen-Centered Health Data Governance in Belgium: Cross-Sectional Survey

**DOI:** 10.3390/healthcare14203461

**Published:** 2026-10-15

**Authors:** Cato van Schyndel, Jochem van Noord, Erik Laes, Karin Hannes, Nathalie Lambrechts

**Affiliations:** 1 Centre for Sociological Research, Faculty of Social Sciences, KU Leuven, Parkstraat 45, 3000 Leuven, Belgium; 2 Flemish Institute for Technological Research (VITO), Boeretang 200, 2400 Mol, Belgium

**Keywords:** personal data spaces, health data, governance, citizen participation, patient empowerment, shared decision-making, data sharing, public perceptions, survey study, digital health

## Abstract

**Highlights:**

**What are the main findings?**

• Support for Personal Data Spaces (PDSs) was conditional and significantly associated with understanding of PDSs, trust in technology, and privacy attitudes.
• Citizens valued control over their data more strongly than transparency and preferred storing medical over lifestyle data. Willingness to share varied by purpose and recipient type, with substantially larger differences associated with recipient type.

**What are the implications of the main findings?**

• PDSs can contribute to meaningful citizen participation particularly when technological features are embedded in governance arrangements that provide real agency, credible safeguards, and demonstrable public value.

**Abstract:**

Background/Objectives: Personal Data Spaces (PDSs) are promoted as infrastructures that strengthen citizen participation in health data governance through transparency and individual control. However, empirical evidence on citizens’ views of PDSs remains limited. This study examined how citizens evaluate *We Are*, a proposed citizen-centered PDS model in Flanders, Belgium, including overall support, preferences for control and transparency, acceptance of different data types and uses, and factors associated with support. Methods: An online survey was conducted among 1041 adults aged 18–79 years in Flanders using quotas for gender, age, education, province, and urbanization. Participants evaluated the *We Are* model and completed measures concerning reasons for support, control and transparency, storage of medical and lifestyle data, and data sharing across purposes and recipient types. Data were analyzed using *t*-tests, linear regression, and mixed models with repeated measures. Results: *We Are* evaluations were ambivalent but slightly positive (M = 2.51 on a 1–4 scale) and did not differ significantly from the scale midpoint. Sociodemographic characteristics explained little variance in support (R^2^ = 0.033), whereas adding understanding and psychographic factors increased variance to R^2^ = 0.238. Respondents valued control more than transparency and were more willing to store medical than lifestyle data (Δ = 0.380, *p* < 0.001). Willingness to share data was higher for both personal and public non-commercial uses than for public commercial uses, whereas personal commercial uses were rated lower. Private recipients were associated with lower willingness to share than public recipients. Conclusions: Support for PDSs was conditional and related more strongly to understanding and psychographic factors than to sociodemographic characteristics. These findings indicate that PDSs should be embedded in governance arrangements that provide real agency, credible safeguards, and demonstrable public value.

## 1. Introduction

Digital health technologies are transforming healthcare by enabling large-scale collection of personal health data through wearables, electronic records, and health apps [1]. These technologies have the potential to contribute to better health outcomes by supporting disease prevention, disease management, and more efficient healthcare delivery [2,3]. Yet the use of health data for research, policy, or commercial innovation raises ethical, legal, and privacy concerns around ownership, consent, security, and misuse of sensitive information [4]. Ensuring data accessibility and reuse while protecting privacy and individuals’ control over their data is therefore an important challenge for data governance [5]. In response, the European Union has introduced regulatory frameworks such as the General Data Protection Regulation (GDPR), the Data Governance Act (DGA), and the European Health Data Space (EHDS) [6,7]. These frameworks seek to harmonize governance, protect individual rights, and empower citizens to exercise control over their data.

Although these frameworks formally aim to empower citizens, evidence shows that participation in health data governance remains limited [8,9]. Governance refers to the actors, rules, and power relations that shape access, sharing, and decision-making [10]. Participation describes the ways citizens can influence these arrangements. Arnstein’s ladder of participation highlights a spectrum from informing and consultation to partnership or citizen control [11]. Our overview of reviews found that, despite generally favorable attitudes toward participation, citizens’ actual involvement in health data governance remains largely limited to informing and consultation rather than decision-shaping roles [9]. At the same time, the review identified that citizens express a desire for more meaningful involvement, valuing transparency, control, and opportunities for both personal and collective benefit [9]. This gap between regulatory ambitions, practice, and expectations risks undermining the perceived legitimacy of health data governance, understood here as public acceptance of how health data are collected, stored, and used. Addressing it requires infrastructures that give citizens real capabilities to check, update, and set preferences for data use, thereby turning abstract rights into practical participation [12,13].

Personal Data Spaces (PDSs) have emerged as a technological response to this challenge. A PDS is a user-centered digital environment in which individuals can store and manage their personal data, including health-related information, and decide under which conditions these data may be accessed or reused by third parties [14,15]. Rather than data being held exclusively by institutions or service providers, PDSs enable individuals to aggregate data from multiple sources, such as healthcare providers, apps, or devices, and to grant or withhold access in a granular and transparent manner [16]. PDSs can be implemented through different technical architectures, ranging from decentralized data pods to platform-based personal data stores, but they share a common emphasis on user control, consent management, and interoperability [17]. Well-known examples include Solid, OpenPDS, and Databox [17]. At the same time, user-controlled data architectures must comply with legal requirements and operate within existing data protection and healthcare regulation, including the GDPR in the European context [18]. Previous research suggests strong public interest in such infrastructures: many citizens view current data handling as problematic and consider PDSs a promising way to regain control over their data and engage more actively in health data governance [16]. In a healthcare context, these capabilities may also be relevant to patient-centered care and shared decision-making (SDM). By enabling individuals to access, manage, and selectively share health data, PDSs may support greater patient autonomy and more active participation in decisions about their care [13].

Despite growing interest in PDSs, several questions remain insufficiently understood. It remains unclear how citizens evaluate PDSs as infrastructures for participation in health data governance rather than merely as privacy-enhancing technologies. Limited evidence exists on which reasons citizens themselves consider most convincing for supporting PDSs. Although both transparency and control are valued, they offer different degrees of influence over health data governance. The ladder of citizen participation developed by Arnstein [11] distinguishes between being informed and having decision-making power, with the latter reflecting a greater degree of citizen influence. Applied to health data governance, transparency enables citizens to understand how their data are used, whereas control additionally allows them to influence decisions about data access and use [8,19]. As PDSs aim to strengthen citizens’ agency in health data governance, we therefore expect that citizens will place greater importance on control than on transparency. It also remains unclear whether citizens are equally willing to store medical and lifestyle data within a PDS. Previous qualitative research found that patients valued sharing medical data with healthcare providers but questioned the added value of sharing lifestyle data [20]. Although both types of data may raise privacy and sensitivity concerns, this finding suggests that citizens might see greater value in storing medical data within a PDS. We therefore expect citizens to be more willing to store medical than lifestyle data. Finally, little is known about how citizens evaluate personal versus public benefit uses within PDSs. Previous research shows that citizens value both the personal benefits that health data use may provide for themselves or their families and its broader benefits for research and society [9]. Research on health data sharing has also highlighted the importance of personal relevance, with citizens expressing greater willingness to share data when the anticipated benefits relate to their own health conditions [21]. Building on this, we reason that personal benefits may be more directly relevant to individuals than benefits directed toward society more broadly, and therefore expect citizens to evaluate personal benefit uses more favorably than public benefit uses within a PDS.

This study addresses these gaps by examining public perceptions of *We Are*, a proposed citizen-centered PDS model in Flanders, Belgium, that integrates participatory governance mechanisms. We assess overall evaluations of the model (H1), reasons for supporting PDSs (H2), preferences for transparency versus control (H3), willingness to store different types of health data (H4), and evaluations of different purposes of data use within a PDS (H5). We hypothesize generally positive evaluations of the model (H1), stronger support for reasons related to personal and public benefits (H2a) and a positive association between endorsement of these reasons and overall support for PDSs (H2b), a stronger preference for control over transparency (H3), greater willingness to store medical than lifestyle data (H4), and more favorable evaluations of personal than public benefit uses of data (H5). Whereas H2 focuses on reasons for supporting PDSs in general, this final hypothesis examines how citizens evaluate concrete purposes of data use within a PDS.

## 2. Materials and Methods

### 2.1. Participant Recruitment and Survey Administration

We used data from an internet-based survey of an opt-in sample of adults aged 18 to 79 in Flanders, Belgium, matched to demographic quotas. A quota-based sampling strategy was applied to approximate the distribution of the Flemish population with respect to gender, age, education, province, and urbanization level. The final sample (N = 1041) reflects these characteristics. The survey was administered online by an external research organization, Indiville, via its Bpact panel, an existing opt-in online panel of participants in Belgium used for survey research on societal topics [22,23]. Data collection took place in January 2024. Participants were invited by email, without disclosure of the survey topic. The number of panel members invited to participate was not available to the researchers, and a response rate could therefore not be calculated. No statistical weighting was applied. Participants were rewarded through the panel’s points-based incentive system redeemable for monetary rewards or charitable donations. As participation relied on an opt-in panel, self-selection bias remains possible and should be considered when interpreting results.

### 2.2. Survey Development and Structure

The survey was developed based on gaps identified in the literature and in collaboration with partners from the *We Are* initiative, including representatives from research institutions, healthcare organizations, general practitioners, and patient organizations (including patient experience experts). Survey items were derived from the literature and iteratively refined in collaboration with these partners. The survey was intended as an exploratory quantitative assessment of public perceptions of *We Are* and forms part of a broader mixed-methods research program in which complementary qualitative work is used to further investigate how citizens evaluate personal data spaces and their role in health data governance.

The survey was designed and pretested for usability, clarity of content, technical functionality, and routing logic with 10 participants. It consisted of several thematic blocks: sociodemographics, health profile, behaviors and experiences, knowledge and attitudes, the *We Are* concept test, and willingness to share data. All items required a response to prevent missing data, and item order within question batteries was randomized. Adaptive questioning ensured that some items appeared only when relevant. Participants could review and revise their answers. The survey was administered in Dutch; the full questionnaire is provided in Supplementary Material S1 and the English translation in Supplementary Material S2.

Participants were introduced to *We Are* through a brief description of a secure personal data vault connecting health information and enabling user-controlled data sharing. The text emphasized explicit consent options and ethical evaluation of potential data recipients and is provided in Appendix A in the original Dutch version and Appendix B in English translation. Participants were asked whether they understood the proposed model (as not all items included in our analyses revolved around the proposed model, all participants were retained in the analyses so that all analyses analyzed the same respondents). Although “personal data vault” was used in the survey for tangibility [13,15,24,25], we use “personal data space (PDS)” throughout the paper to align with scholarly terminology.

### 2.3. Data Protection and Quality Control

Responses were automatically captured, anonymized, and stored on a secure server managed by the survey administrator. After fieldwork, anonymized data were transferred to the research institution. Duplicate responses were prevented using unique panel identifiers. Data screening procedures included the identification of incomplete submissions, assessment of response durations, and evaluation of response patterns (e.g., minimal variation across Likert-scale items). Response duration was considered together with variation across scale items, rather than applying a fixed exclusion threshold based solely on completion time. Implausibly fast submissions or otherwise questionable responses were reviewed on a case-by-case basis and excluded where appropriate. This review was conducted by Indiville before delivery of the final dataset, and the exact number of responses excluded during this process was not available to the researchers. All survey items required a response, so no questions were left unanswered. However, several questions included an “I don’t know” answer category, which was coded as NA for analysis. For the fraud victimization variable, respondents who selected “prefer not to answer” were excluded from analyses involving this variable. The gender category X was included in all analyses but not reported in tables due to the very small number of observations. No IP addresses were collected.

### 2.4. Ethical Considerations

Ethical approval was obtained from the Ethics Committee of University Hospital of Antwerp (UZA)/UAntwerp (BUN300201940638). Informed consent was obtained from all participants prior to participation, in accordance with the approved ethical dossier. Participation was voluntary, and respondents were free to discontinue the survey at any time without consequence. Participants provided informed consent to participate in the study, including the use of their pseudonymized data for scientific research and reporting of aggregated results.

### 2.5. Measures

#### 2.5.1. General Evaluation of PDSs

To examine how citizens evaluate PDSs as instruments for participation in health data governance (H1), we measured participants’ overall assessment of *We Are* as a concrete PDS model. After reading the *We Are* description, participants evaluated the concept on four dimensions: attractiveness, personal relevance, credibility, and trustworthiness. These dimensions are consistent with technology acceptance research, particularly the Unified Theory of Acceptance and Use of Technology (UTAUT), which highlights perceived usefulness and relevance, as well as confidence in a system and its institutional embedding, as core drivers of acceptance [26]. In our context, attractiveness and personal relevance reflect benefit expectations akin to performance expectancy, whereas credibility and trustworthiness capture confidence in the PDS and its governance. To examine the underlying structure, we conducted exploratory factor analyses. Parallel analysis suggested two dimensions (attractiveness and personal relevance versus credibility and trustworthiness), and an exploratory factor analysis with oblimin rotation confirmed this pattern with a relatively strong correlation between the two factors (*r* = 0.69; Eigenvalue_dimension1_ = 1.50; Eigenvalue_dimension2_ = 1.36). Results with respect to our hypotheses were consistent when modeled as one or two factors (some differences between the scales were found for the control variables predicting PDS support and for the reasons for supporting PDSs. We report these differences in Appendix C and Appendix E.). Hence, for brevity, we report results only for a scale where the four items were combined into a single scale (Cronbach’s alpha = 0.84), with higher scores indicating a more positive evaluation of *We Are*. When results differ between the two sub-scales, we report these in footnotes. Results on analyses with the two separate scales are listed in Appendix C.

#### 2.5.2. Reasons for Supporting PDSs

To understand why citizens support PDSs and which kinds of benefits they find most convincing (H2a), participants were asked which reasons most persuaded them to use *We Are*. The question presented thirteen possible reasons covering transparency, control, convenience, privacy protection, personal health management, and broader social benefits (“Below is a list of different reasons to use We Are. Can you indicate the reasons that most convince you to use We Are? You can select a maximum of three reasons”). An additional option allowed respondents to indicate “none of the above”. Each reason was coded 0 (not selected) or 1 (selected), with participants allowed to choose up to three. We then calculated the percentage endorsing each reason and examined the difference in the means of overall support for *We Are* between those who do and do not endorse the reason. This allowed us to distinguish between reasons that were frequently cited but weakly associated with support and those that were less common but strongly predictive of higher support (H2b).

#### 2.5.3. Participation Preferences in PDSs

To assess whether citizens prioritize control or transparency in the context of PDSs (H3), we used three attitudinal items from a broader battery on data sharing and privacy. Transparency was measured with two items capturing different domains: one referring specifically to personal health data (“I only share personal health data if I know what they will be used for”) and one referring to personal data in general (“Before I share my personal data, I want to know what they will be used for”). A third item captured control (“I find it important to have control over whom I share which data with”). All items were rated on a five-point Likert scale from strongly disagree to strongly agree. The two transparency items were analyzed separately to account for potential differences between health-specific and general data sharing preferences.

#### 2.5.4. Types of Health Data Stored in PDSs

To examine how citizens evaluate storing different types of health-related data in PDSs (H4), participants reported to what extent they would like to store various types of health data in the *We Are* data vault (“Please indicate below to what extent you would like to store the following personal data about your health in the We Are data vault”). Each item was rated on a four-point scale: definitely not, rather not, somewhat, or definitely yes. To identify broader data factors, we conducted a parallel analysis followed by factor analysis. Results consistently indicated two factors corresponding to medical data (e.g., blood test results, hospital admissions, medication use; Eigenvalue = 7.44) and lifestyle data (e.g., diet, exercise, sleep; Eigenvalue = 3.19). These two categories were used in subsequent analyses to compare willingness to store medical versus lifestyle data in PDSs.

#### 2.5.5. Intended Purpose of Data Use in PDSs

To investigate how citizens evaluate different purposes for sharing data stored in PDSs (H5), we presented twelve short vignettes describing concrete data sharing scenarios. Each vignette systematically varied both the intended purpose of data use (personal versus public benefit and commercial versus non-commercial) and recipient type (public versus private organization). For example, one scenario described sharing data about your purchasing behavior regarding medicines with a pharmaceutical company to support the marketing of their products (public benefit, commercial), while another described sharing lifestyle data with a family doctor for tailored care (personal benefit, non-commercial). As prior research identifies recipient type (public versus private organization) as an important determinant of data sharing preferences, it was included as a control variable to isolate the effect of intended purpose of use [27]. Willingness to share in each scenario was rated on a five-point scale from not at all willing to quite willing, with an additional “I do not know” option. To examine whether responses clustered into broader evaluative patterns, we conducted a parallel analysis followed by factor analysis. Although initial results suggested three factors, theoretical considerations indicated that the scenarios most clearly reflected two cross-cutting categories inherent to the vignette design: intended purpose of use and recipient type. In subsequent analyses, we focused on differences in willingness to share across purposes of use, while statistically controlling for recipient type.

### 2.6. Control Variables

We included six demographic (gender, age, educational level, main activity, general self-rated health, fraud victimhood) and six psychographic (internet knowledge, pro-privacy attitude, pro-privacy behavior, trust in technology, privacy fears due to technology, technological adoption) control variables. Pro-privacy attitude was assessed with a battery of items about data sharing and app/website permissions (e.g., webcam use). Parallel analysis suggested six factors, but most were difficult to interpret or contained only a single item. A follow-up factor analysis (oblimin rotation) produced two clearer dimensions: one capturing general pro-privacy attitudes and another reflecting willingness or reluctance to share specific data (Eigenvalue_dimension1_ = 2.87; Eigenvalue_dimension2_ = 2.58). The factor loadings are reported in Appendix D. For the analysis, we used the first dimension focusing on attitudes (combining items b, d, i, j, n, q, s, where items b, i, j, q were reverse-scored). The second dimension with its focus on specific data sharing was deemed less relevant and, more importantly, overlaps theoretically with the (in our view more appropriate) pro-privacy behavior scale presented below. The scale was calculated as the mean of all items with loadings above 0.4 (Cronbach’s alpha = 0.77). Pro-privacy behavior was measured with 17 possible privacy-protective practices (e.g., using anti-tracking software, Virtual Private Networks (VPNs), or removing cookies). Respondents indicated whether they had carried out each practice in the past three months. Items were coded 0/1 and averaged into a single score, with higher values reflecting more frequent protective behavior (Cronbach’s alpha with polychoric correlations = 0.87).

### 2.7. Analytical Approach

We applied different statistical techniques depending on the type of outcome. General evaluation of PDSs (H1) and participation preferences in PDSs (H3) were analyzed using linear regression and *t*-tests. Reasons for supporting PDSs (H2) were analyzed by examining endorsement frequencies and mean differences in overall support between endorsers and non-endorsers. Types of health data stored (H4) and intended use of data (H5) involved repeated observations from the same individuals (since each respondent evaluated multiple scenarios or data types). To account for this nesting, we used mixed models with repeated measures (MMRMs). This approach allows the analysis to capture correlations between responses from the same participant. We specified an unstructured covariance matrix, which allows the model to freely estimate the correlations among a participant’s responses across different items rather than imposing a fixed pattern. Analyses were conducted using the *mmrm* package in R (Release 4.5.0) [28]. Hypotheses were evaluated based on the direction and statistical significance of estimated effects. Statistical significance is based on *p*-values that are not corrected for multiple comparisons, unless specified. In that case, we used the Holm step-down procedure [29]. All code to reproduce our analyses is available at the Open Science Foundation (https://osf.io/s2y5j/overview?view_only=55daad23c2034f759998cc2c539f7996, accessed on 4 October 2026), data are available on request.

### 2.8. Reporting Guidelines

We followed the Checklist for Reporting of Results of Internet E-Surveys (CHERRIES) to ensure transparency and methodological rigor in reporting [30]. The completed checklist is provided in Supplementary Material S3.

## 3. Results

### 3.1. Descriptive Statistics

Table 1 summarizes the sample (N = 1041). The opt-in sample was matched to the distribution of residents of Flanders, Belgium, aged 18–79 with respect to gender, age, educational level, province, and level of urbanization. Men and women are almost evenly represented (499 and 541; one respondent identified as X). Age and education are well distributed across categories, and the largest activity groups are private-sector employees and retirees. Average self-rated health is moderately high (Mean = 3.74, Standard Deviation = 0.80). About one in five respondents report fraud victimhood in the household (21.9%). Psychographic indicators show relatively high internet knowledge, generally pro-privacy attitudes with less frequent privacy-protective behavior, moderate trust in technology alongside notable privacy fears, and moderate technology adoption.

### 3.2. General Evaluation of PDSs

We hypothesized (H1) that citizens would evaluate the PDSs positively overall. On the 1-to-4 support scale, the mean score for *We Are* was 2.51, which is not significantly higher than the midpoint of the scale (M = 2.5, *t*(1040) = 0.703, *p* = 0.241). H1 was therefore not supported. The median is 2.63 with more respondents (N = 576) scoring above the midpoint than below or at the midpoint (N = 465).

To examine predictors of support (Table 2), we estimated two linear regression models. In Model 1, which included only sociodemographic factors, education was the only significant correlate: secondary- and tertiary-educated respondents evaluated *We Are* more positively than primary-educated respondents. In Model 2, which added understanding of *We Are* and psychographic variables, model fit improved markedly from R^2^ = 0.033 to R^2^ = 0.238, indicating that knowledge and attitudes explain substantial variance in support. Understanding the system was strongly and positively associated with support and the education effects observed in Model 1 were no longer significant. Trust in technology was positively related to support, whereas stronger pro-privacy attitudes and privacy fears regarding technology were negatively associated. Internet knowledge, privacy-protective behavior, and technology adoption were not significantly related to support (when modelled with the separate *We Are*-evaluation scales, privacy behavior is significant with Scale 1 (attractiveness and personal relevance) but not Scale 2 (credibility and trustworthiness); privacy fears technology is significant with Scale 2 but not Scale 1; technology adoption is significant with Scale 1 but not Scale 2. All other results were the same. Full results are reported in Appendix C).

### 3.3. Reasons for Supporting PDSs

We hypothesized (H2) that reasons relating to both personal and public benefits would be frequently endorsed and associated with stronger support. Table 3 reports the percentage of respondents endorsing each reason and the difference in mean support for *We Are* between those who endorse the reason and those who do not. Participants were only allowed to pick a maximum of three reasons. Hence, % agree indicates to what extent these reasons belonged to the three most important reasons for using *We Are*. The most frequently selected reasons were that data should be used only with explicit consent (38%), keeping personal health data in one place (37%), and guaranteed privacy (30%).

The reasons most strongly associated with higher support were contributing to better health policy, keeping data in one place, and receiving better healthcare oneself. All mean differences were statistically significant at *p* < 0.05, except tailored services and, when correct for multiple comparisons, “I can contribute to better services and products”. These findings provide partial support for H2: both personal and public benefit-related reasons were associated with higher PDS support, but practical and system features were also among the strongest predictors (when modelled with the separate *We Are*-evaluation scales, “I retain control over my personal health data” and “By sharing my personal health data, I can contribute to better services and products” are insignificant with scale 2 (credibility and trustworthiness). All other results are the same. Full results are reported in Appendix E).

### 3.4. Participation Preferences in PDS

We hypothesized (H3) that citizens would value control more strongly than transparency. Results from paired *t*-tests indicate that respondents rated control (M = 4.16, SD = 0.78) significantly higher than transparency for both general personal data (M = 3.83, SD = 0.85, b = 0.33, 95% CI [0.27, 0.39], *t*(1040) = 10.37, *p* < 0.001) and personal health data (M = 3.83, SD = 0.87, b = 0.33, 95% CI [0.27, 0.39], *t*(1040) = 12.47, *p* < 0.001), indicating a consistent preference for the ability to decide with whom data are shared over simply being informed about use.

To explore whether this preference varied across background characteristics, we estimated a regression model for the difference between transparency for general personal data and control and personal health data and control (Table 4). The explained variance was low in both analyses (R^2^ = 0.026 and 0.019 respectively), showing that sociodemographic and psychographic factors account for only a small part of the preference gap. For general data sharing, stronger privacy-protective behavior was associated with a stronger preference for control (*p* < 0.05). For health data, stronger privacy fears were associated with a stronger preference for control (*p* < 0.05). No other predictors were significant. Overall, these findings support our hypothesis (H3) that people indicate a stronger preference for a sense of control over data sharing than for transparency on what happens with data.

### 3.5. Types of Health Data Stored in PDSs

We hypothesized (H4) that medical data would be seen as more acceptable to store in a trusted PDS than lifestyle data. Using MMRMs, we compared willingness to store medical and lifestyle data in *We Are*. Willingness to store medical data was significantly higher than for lifestyle data (Δ = 0.380, Standard Error = 0.020, *p* < 0.001). When genomic data, which differ from other medical data due to their particularly sensitive nature, were excluded from the medical category, the difference increased further (Δ = 0.457, Standard Error = 0.021, *p* < 0.001) (this analysis was not preregistered). Interactions between data type and demographic characteristics were not significant, indicating that the medical–lifestyle gap did not differ systematically across subgroups. The full model, including the demographic control variables, is reported in Appendix F. These findings support H4: respondents found the storage of medical data more acceptable than lifestyle data.

### 3.6. Intended Purpose of Data Use in PDSs

We hypothesized (H5) that personal benefit uses of data would be seen more favorably than public benefit uses. We estimated an MMRM predicting willingness to share across scenarios that varied by intended purpose of data use, while controlling for recipient type (Table 5). The full model, including all control variables, is reported in Appendix G, and the classification of each vignette by intended purpose and recipient type is provided in Appendix H.

Relative to the reference category, for commercial public benefit (for example, sharing data on medicine purchases with a pharmaceutical company for marketing), willingness to share was lower than for personal benefit commercial uses (for example, sharing data with a commercial app to obtain a discount). When compared to public benefit non-commercial uses (for example, sharing hospital experience data with the hospital to improve care), willingness to share for personal benefit non-commercial uses (for example, sharing data in a health vault with emergency services) was not significantly different (b = 0.005, Standard Error = 0.015, *p* = 0.716). These findings provide no support for H5: personal benefit use was not rated more favorably than public benefit use. The differences associated with the intended purpose of data use were considerably smaller (|b| ≤ 0.074) than the difference associated with recipient type (b = −0.670), which indicates that willingness to share was more strongly associated with who received the data than with its intended purpose.

## 4. Discussion

Drawing from a survey of 1041 Flemish citizens recruited through an opt-in panel and matched to demographic quotas, this study examined how individuals evaluate *We Are*, a PDS designed to enhance transparency and control in health data governance. The aims were fivefold: to assess overall evaluations of the PDS (H1); to identify which reasons for supporting PDSs, including personal or public benefits rationales, are most convincing (H2a and H2b); to compare the importance citizens attach to control versus transparency (H3); to examine willingness to store different types of health data, particularly medical versus lifestyle data (H4); and to assess how citizens evaluate concrete purposes of data use within a PDS and whether commercial versus non-commercial contexts shape these evaluations (H5).

### 4.1. Implications for Research, Policy, and Practice

#### 4.1.1. Beyond Demographics Toward System Understanding and Trust

Regarding H1, the mean score was 2.51 on a 1-to-4 scale and did not differ significantly from the midpoint, meaning that H1 was not supported. Descriptively, evaluations were ambivalent with a slight positive tendency. This is more cautious than findings by Maes et al., who report more clearly favorable attitudes toward PDSs in a similar Flemish context [16]. This difference likely reflects the broader scope of the present study, which examines PDSs not only as a tool for data control but as infrastructures for active citizen participation in health data governance. This suggests that while PDSs are seen as potentially promising instruments for strengthening control and transparency in health data governance, they also elicit reservations that temper stronger endorsement. A smaller group evaluated *We Are* negatively, consistent with diffusion of innovation theory, which holds that new technologies are rarely adopted uniformly: early adopters tend to be enthusiastic, whereas others remain cautious until benefits become tangible [31]. As PDSs become visible in practice, early adopters may therefore play an important role in demonstrating their value and familiarizing others with their use.

Education initially predicted higher support, but this effect disappeared once understanding and psychographic variables were included, indicating that evaluations cannot be explained by sociodemographic characteristics alone. This aligns with research showing that sociodemographic segmentation offers only a limited picture of public responses, whereas psychographic approaches better capture the motives underlying acceptance [32]. In this study, understanding of the *We Are* model emerged as a strong predictor of support, highlighting that acceptance depends on whether citizens grasp how the system functions and what safeguards it offers. Previous research suggests that comprehension challenges may vary across sociodemographic groups, implying that older adults or lower-educated citizens may require different forms of communication and support than younger or more digitally experienced groups [33]. Public communication about PDSs should therefore be tailored to make participation intelligible across diverse segments of the population [34].

The prominence of understanding also helps explain the cautious nature of support. When data infrastructures remain hypothetical, citizens may struggle to imagine how they would operate or affect their lives, and their support may depend on who they believe would host and govern the data space [35]. Previous research shows that public organizations are generally trusted more than private ones, thereby shaping willingness to share data [36,37].

Trust in technology was positively associated with support, whereas stronger privacy concerns and fears reduced it. By contrast, indicators of digital experience, such as internet knowledge, privacy-protective practices, and technology adoption, were unrelated to PDS evaluation. This suggests that support is shaped less by what people do online than by how they perceive the balance between privacy and benefit, reflecting both the intention–behavior gap and the privacy paradox [38].

These findings show that support for PDSs cannot be explained by digital competence or routine online behavior alone. Instead, it is grounded in broader orientations of confidence, skepticism, and perceived institutional credibility. This reliance on attitudes also points to a potential fragility: public views on data governance can shift quickly in response to media coverage, data breaches, or institutional failures. As Meier et al. show, such shifts can outweigh established habits and trigger significant governance reforms [39]. Building durable confidence in PDSs therefore requires not only robust technical design but also clear accountability and transparent communication. More fundamentally, these findings indicate that the key challenges raised by PDSs are not primarily technical. While PDS technologies can enable secure data storage, transparency, and control, they cannot determine whose interests are prioritized, which data uses are legitimate, or how collective benefits are safeguarded. These are inherently governance questions. Citizens’ evaluations of *We Are* thus reflect not only the perceived capabilities of the technology, but also normative expectations about acceptable data use, control, and safeguards in practice.

#### 4.1.2. Tangible Benefits Require Credible Safeguards

Regarding H2 and H5, we examined both the reasons citizens give for supporting PDSs and how they evaluate concrete purposes of data use within a PDS. This dual approach allowed us to assess not only which reasons were most frequently cited, but also which ones were most strongly associated with overall support for the *We Are* model. The findings show that both personal and public benefit arguments play an important role. Citizens found PDSs particularly convincing when they promised to improve their own healthcare or contribute to better healthcare policy. This dual orientation toward individual and collective outcomes aligns with previous research indicating that willingness to share health data often reflects solidarity rather than narrow self-interest [8,40]. Citizens thus evaluate PDSs not only in terms of personal gain, but also by their perceived societal value.

Evaluations of concrete data-use purposes provided a more nuanced picture. Respondents granted legitimacy to both personal and collective uses of data, consistent with earlier studies [36,41,42,43,44]. However, personal benefits were not valued uniformly. Personalization, often highlighted in digital health discourse as a key advantage, contributed little to support when it resembled consumer targeting rather than genuine health improvement. Tailored lifestyle advice or commercial offers may be perceived as marketing rather than care, echoing findings that high levels of personalization can evoke discomfort and distrust rather than engagement [45]. By contrast, personalized uses that demonstrably improved treatment or emergency care were seen as meaningful. These distinctions suggest that meaningful participation is not reducible to individual benefit alone, but that it depends on whether benefits are perceived as legitimate within a broader moral economy. The findings also suggest that evaluations depend on the specific purpose and context of data use, rather than on whether the benefit is personal or collective alone. As Braun and Hummel argue, legitimacy in data governance rests on values of solidarity, fairness, and trustworthiness rather than on individual rewards [46].

At the same time, system-related features were among the most frequently cited reasons for supporting PDSs. Respondents emphasized consent, privacy protection, and the ability to keep data in one place. These findings suggest that such features may be important conditions for citizens’ acceptance of PDSs, although the present study cannot establish them as necessary conditions for participation. This interpretation aligns with studies showing that credible safeguards are a precondition for acceptance, while differences in support depend more strongly on perceived value and purpose [8,37,44,47,48]. Without such safeguards, even compelling promises of better care or policymaking lose credibility.

Taken together, these findings suggest that combining tangible benefits with credible safeguards may contribute to citizens’ acceptance of PDSs. Benefits may generate perceived value, while safeguards may generate confidence that data will be used appropriately. Together, these elements could provide a basis for PDSs to function as credible instruments for meaningful citizen participation in health data governance, although this requires further investigation in practice.

#### 4.1.3. Complementary Roles of Transparency and Control in Participation

Regarding H3, we examined whether citizens prioritize transparency or control because these dimensions reflect different modes of participation in health data governance. Transparency concerns being informed about data practices, whereas control entails the ability to influence decisions about data access and use. Distinguishing between the two is essential for assessing whether PDSs are perceived mainly as informational tools or as infrastructures for meaningful participation. Citizens valued control more strongly than transparency across both general and health data contexts. Respondents prioritized the ability to decide who could access their data over simply being informed about how data are used. However, both dimensions received relatively high scores, suggesting that citizens do not view transparency and control as competing, but as complementary. Transparency provides awareness of data practices, while control enables action on that information by allowing individuals to shape data-sharing decisions [8,47].

The emphasis on control was particularly pronounced among respondents with stronger privacy fears and more privacy-protective behaviors. For these citizens, control may function as a protective mechanism, offering reassurance in contexts where trust is fragile [27,49,50]. The models explained relatively little variance in preferences for control versus transparency, and the sociodemographic characteristics included in the analyses were not significantly associated with these preferences. This does not rule out differences associated with other individual or contextual characteristics. Nevertheless, the overall preference for control observed in this study is consistent with previous research showing that control is widely valued across populations [51].

Taken together, these findings suggest that respondents value transparency but place greater importance on having control over data access and use. Conceptually, combining transparency with mechanisms that allow citizens to act on that information may provide a stronger basis for participation than transparency alone. Whether this translates into meaningful participation in operational PDSs requires further investigation.

The greater importance respondents placed on control may also be relevant to patient-centered care and shared decision-making (SDM). Patient empowerment involves not only access to information, but also the agency to make choices and participate in decisions about care [52]. In the context of PDSs, control over personal health data may give patients a more active role in determining what information is available when decisions about their care are made [13]. However, PDSs do not themselves constitute SDM, which involves patients and healthcare professionals making clinical decisions together [53]. Rather, the control provided by PDSs may support SDM by giving patients greater influence over the information that enters this decision-making process. Future research could therefore examine whether PDSs support such involvement when used in clinical practice.

#### 4.1.4. Data Relevance and Context in Judging Acceptability

Regarding H4, we examined how citizens evaluate the acceptability of storing different types of health-related data in a PDS, focusing on differences between medical and lifestyle data. Citizens made clear distinctions between data types. Medical data, such as doctor visits, medication records, or test results, were judged more suitable for inclusion than lifestyle data collected through apps or wearables. This suggests that willingness to store data in PDSs is shaped not only by the type of data itself, but also by whether its use is perceived as legitimate and meaningfully connected to healthcare. In line with previous research, respondents appeared more willing to accept medical data sharing when the healthcare purposes and safeguards surrounding data use were considered appropriate and trustworthy [8,36,37,42,47,48,54,55,56].

By contrast, lifestyle data such as sleep patterns, diet, or physical activity elicited greater hesitation. Rather than relating directly to diagnoses or treatment, such data expose everyday behaviors that people may prefer to keep private and may be perceived as intrusive, commercially motivated, or of limited relevance to healthcare [57]. This perception of limited relevance or potential misuse may help explain their lower acceptability. As prior research indicates, acceptance depends on whether citizens perceive a clear and trustworthy link between data use and health outcomes [58].

Overall, these findings show that decisions about which data belong in a PDS are shaped by perceived relevance, purpose, and legitimacy within a health context. Technical protection alone is insufficient to secure public acceptance; it must be accompanied by transparent communication about why specific data are collected, how they support health goals, and what safeguards prevent misuse.

### 4.2. Strengths and Limitations

This study makes several contributions to the understanding of citizen participation in health data governance through PDSs. A first strength is the use of a large survey sample of 1041 respondents matched to the Flemish population across key sociodemographic characteristics, including age, gender, educational level, province, and urbanization level. This provides broad demographic coverage within the Flemish context. Second, the survey was carefully developed and tested for clarity, usability, and completeness, minimizing missing data and ensuring robust measurement. Third, the use of the *We Are* model, co-developed with patient experience experts, allowed the survey to present a concrete and coherent governance scenario rather than a purely technical or abstract description of data-linking technology, thereby increasing ecological validity.

At the same time, the study has limitations. Participation relied on a voluntary online panel, which may have attracted individuals more inclined to engage with surveys or with stronger opinions on societal issues, introducing potential self-selection bias. The sample may therefore differ from the wider Flemish population on characteristics not captured by the demographic quotas, and the findings should not be interpreted as representative of the wider population. The survey was administered online, which could underrepresent individuals with lower digital literacy or limited internet access, even though demographic quotas were applied. In addition, the analysis does not specifically differentiate perspectives of individuals with more intensive or sustained interactions with healthcare systems, such as those with chronic conditions, whose views on health data governance may differ. Future research could examine these perspectives in more depth. Moreover, the findings are specific to Flanders and may not fully generalize to other cultural or regulatory contexts where attitudes toward data sharing and governance differ. Future research could extend this to mobile and cross-border populations, for whom PDSs may be particularly relevant because they can support citizen-controlled access to health data across healthcare systems and national borders [59]. A further limitation is that the *We Are* model was introduced through a short text. Despite being co-developed with patient experience experts, such a concise description may not have been fully understood by all respondents, potentially limiting the depth of their evaluations. Moreover, the survey contained mostly positively framed items, which may have tilted responses toward more favorable perceptions of PDSs and offered limited opportunity for respondents to articulate critical views. Future research should complement survey findings with qualitative methods, such as interviews or focus groups, to explore underlying motivations, capture reservations in greater depth, and add nuance to the observed patterns. In addition, the analysis of reasons for supporting PDSs relied on items that were not evenly distributed across constructs: some aspects (e.g., control) were captured by multiple items, whereas others (e.g., collective benefit) were represented by a single item, which may have influenced the relative strength of observed associations. The study also relied on self-reported attitudes and intentions, which may not translate directly into behavior once PDSs are implemented. Future research could refine the measures and test them across different populations and PDS designs. Finally, the cross-sectional design prevents conclusions about causality: while associations between attitudes and support were observed, longitudinal or experimental studies are needed to determine how these factors influence participation over time.

Together, these strengths and limitations suggest that the findings provide a valuable but context-bound contribution. They offer an evidence base for policy and practice in Flanders and comparable settings, while also pointing to the need for future research that broadens the scope across populations, methods, and real-world PDS implementations and that examines how citizens envision the role of PDSs in their future health data practices and what conditions they consider necessary for such systems to become feasible, appropriate, meaningful, and effective.

## 5. Conclusions

This study examined how citizens in Flanders, Belgium, evaluate PDSs as infrastructures for participation in health data governance. Overall evaluations were mixed, with a slight positive tendency, suggesting that PDSs are seen as a potentially plausible way to strengthen control and transparency, albeit with reservations. Support was significantly associated with understanding of PDSs, trust in technology, and privacy attitudes. Respondents reported greater willingness to store medical than lifestyle data in a PDS, potentially reflecting doubts about their necessity and concerns about intrusiveness. Willingness to share also varied by purpose and recipient type, with both personal and public non-commercial uses rated more favorably than public commercial uses, while personal commercial uses were rated less favorably and private recipients were associated with lower willingness to share than public recipients. Across domains, control consistently mattered more than transparency alone. Respondents expressed a stronger preference for being able to decide who accesses which data and for what purpose than for being informed about data use alone. Taken together, these findings suggest that PDSs have the potential to move participation in health data governance beyond merely informing or consulting citizens by providing greater control over how personal health data are accessed and used. While PDS technologies can technically enable transparency and control, their potential contribution to meaningful participation may be strengthened by governance arrangements that provide real agency, protect privacy, and demonstrate clear public value. As this study measured self-reported evaluations and intentions rather than actual data-sharing behavior, future research should examine whether these preferences are reflected in practice when citizens interact with operational PDSs. Future research should also examine how these patterns hold across different cultural and regulatory contexts and across diverse PDS designs. Survey-based evidence should be complemented with qualitative approaches to explore how citizens themselves envision the role of PDSs in their future health data practices and what conditions they see as necessary for such systems to become feasible, appropriate, meaningful, and effective.

## Figures and Tables

**Table 1:** Descriptive statistics of the study sample.

Item	N	Mean/%	Standard Deviation	Median	Min	Max
Gender	1041			2	1	3
Male	499	47.9%				
Female	541	52.0%				
X ^a^	1	0.1%				
Age	1041			2	1	4
≤34	267	25.6%				
35–49	258	24.8%				
50–64	308	29.6%				
65+	208	20.0%				
Education	1041			2	1	3
Primary educated	161	15.5%				
Secondary educated	467	44.9%				
Tertiary educated	413	39.7%				
Main activity	1041			3	1	7
Employee (private)	330	31.7%				
Employee (public)	136	13.1%				
Independent	64	6.1%				
Retired	275	26.4%				
Work disabled	78	7.5%				
Student	91	8.7%				
Other	67	6.4%				
General self-rated health	1041	3.738	0.801	4	1	5
Fraud victim	1024	0.219	0.414	0	0	1
Internet knowledge	1041	7.229	1.618	7	0	10
Privacy attitude	1041	3.741	0.647	3.714	1.857	5
Privacy behavior	1041	0.373	0.211	0.353	0	1
Trust in technology	1041	3.392	0.955	3	1	5
Technology fears privacy	1041	3.649	1.037	4	1	5
Technology adoption	1041	2.736	0.772	2.750	0	5
Willingness to store medical data	1041	2.833	0.833	2.900	1	4
Willingness to store lifestyle data	1041	2.123	0.779	2.200	1	4
General support for *We Are*	1041	2.513	0.592	2.625	1	4

^a^ While included in all analyses, this category is omitted in all further tables for brevity, as estimates based on a single respondent do not allow for generalization to a wider population.

**Table 2:** Results from linear regression on general evaluation of PDSs.

	Model 1 B (SE)	Model 2 B (SE)
(Intercept)	2.348 *** (0.126)	1.623 *** (0.197)
Female (ref. = Male)	−0.040 (0.039)	0.046 (0.036)
Age (ref. = ≤34)		
35–49 years	−0.093 (0.059)	−0.066 (0.052)
50–64 years	−0.086 (0.063)	0.018 (0.058)
65 + years	−0.113 (0.088)	−0.015 (0.081)
Education (ref.= Primary educated)		
Secondary educated	0.134 * (0.058)	0.042 (0.052)
Tertiary educated	0.244 *** (0.068)	0.101 (0.062)
Main activity (ref.= private employee)		
Public employee	0.023 (0.060)	−0.049 (0.054)
Independent	0.025 (0.081)	−0.008 (0.072)
Retired	0.039 (0.072)	0.085 (0.064)
Work disabled	0.038 (0.081)	0.060 (0.073)
Student	0.024 (0.081)	0.040 (0.072)
Other	0.033 (0.085)	0.052 (0.076)
General self-rated health	0.020 (0.025)	−0.015 (0.022)
Fraud victim	0.039 (0.045)	0.030 (0.040)
Understands *We Are*		0.251 *** (0.022)
Internet knowledge		−0.010 (0.012)
Privacy attitude		−0.104 *** (0.030)
Privacy behavior		0.039 (0.095)
Trust in technology		0.146 *** (0.021)
Privacy fears technology		−0.041 * (0.017)
Technology adoption		0.045 + (0.027)
N	1024	1024
R^2^	0.033	0.238

Note: Coefficients are unstandardized, standard errors in brackets. *** *p* < 0.001, * *p* < 0.05, + *p* < 0.10.

**Table 3:** Agreement with reasons for using *We Are* and its relationship with support for *We Are*.

Reason for Using *We Are*	% Agree	Difference in *We Are*-Support (%)
I receive clear information about who uses my personal health data, for how long, and for what purpose	18.8 (1.2)	12.7 (4.7)
As a user of *We Are*, I can help decide which organizations can access the data vaults	14.8 (1.1)	22.8 (5.1)
I can keep all my personal health data in one place and thus maintain an overview	36.6 (1.5)	28.4 (3.7)
I don’t have to re-enter my personal health data every time it is needed for different applications	18.5 (1.2)	14.6 (4.7)
I can easily access my personal health data	18.8 (1.2)	15.9 (4.7)
My personal health data can only be used after I give my consent	38.3 (1.5)	16.3 (3.7)
The privacy of my personal health data is guaranteed	30.0 (1.4)	20.9 (4)
I retain control over my personal health data	27.2 (1.4)	11.8 (4.1)
I become more aware of my own health because I have a good overview of my personal health data	7.4 (0.8)	22.6 (7)
By sharing my personal health data, I can contribute to better services and products	4.4 (0.6)	19.4 (8.9)
By sharing my personal health data, I can receive better healthcare myself	14.0 (1.1)	26.5 (5.2)
By sharing my personal health data, I can contribute to better healthcare policy for everyone	10.3 (0.9)	32.5 (6)
I can receive tailored services in exchange for sharing my personal health data	4.7 (0.7)	4.5 (8.7)

Note: Standard error in brackets. Respondents were allowed to pick a maximum of three reasons. Differences in the last column result from a bivariate regression with the *We Are*-support scale and indicate the difference between those who did and did not pick this reason. All differences are significant (*p* < 0.05), except for the last reason. When corrected for multiple comparisons “By sharing my personal health data, I can contribute to better services and products” is also not significant.

**Table 4:** Results of regression analysis of the difference score of preference for control–preference for transparency.

	Control Versus Transparency on General Data Sharing	Control Versus Transparency on Health Data Sharing
(Intercept)	0.071 (0.328)	−0.149 (0.274)
Respondent-level		
Female (ref. = Male)	−0.001 (0.070)	0.025 (0.058)
Age (ref. = ≤34)		
35–49 years	0.060 (0.102)	0.048 (0.085)
50–64 years	0.079 (0.112)	0.006 (0.093)
65+ years	0.168 (0.155)	−0.059 (0.129)
Education (ref. = Primary educated)		
Secondary educated	0.089 (0.101)	0.058 (0.085)
Tertiary educated	−0.089 (0.120)	0.085 (0.100)
Main activity (ref. = private employee)		
Public employee	−0.099 (0.105)	−0.070 (0.088)
Independent	−0.134 (0.141)	−0.081 (0.118)
Retired	−0.062 (0.126)	−0.073 (0.105)
Work disabled	0.088 (0.142)	−0.093 (0.118)
Student	−0.032 (0.141)	−0.185 (0.118)
Other	0.101 (0.148)	0.064 (0.124)
General self-rated health	0.057 (0.044)	0.039 (0.037)
Fraud victim	0.095 (0.078)	0.026 (0.065)
Internet knowledge	0.008 (0.024)	−0.004 (0.020)
Privacy behavior	0.405 * (0.169)	−0.161 (0.141)
Trust in technology	−0.040 (0.042)	0.043 (0.035)
Privacy fears technology	0.018 (0.034)	0.062 * (0.028)
Technology adoption	−0.060 (0.052)	0.010 (0.044)
N (respondents)	1024	1024
R^2^	0.026	0.019

Note: Coefficients are unstandardized, standard errors in brackets. * *p* < 0.05. Positive scores indicate a stronger preference for control over transparency. Other regression models included attitude toward privacy, which is omitted here, since the two transparency items are part of the scale that forms this attitude.

**Table 5:** Results of mixed models with repeated measures analysis on preferences of data use.

	Model
(Intercept)	2.745 *** (0.195)
Item-level	
Intended purpose of data use (ref. = Public benefit commercial)	
Public benefit non-commercial	0.062 ** (0.022)
Personal benefit commercial	−0.074 *** (0.020)
Personal benefit non-commercial	0.068 *** (0.019)
Data recipient type	
Private receiver (ref. = public receiver)	−0.670 *** (0.018)
N (items)	11,823
N (respondents)	1011
R^2^	0.263

Note: Coefficients are unstandardized, standard errors in brackets. *** *p* < 0.001, ** *p* < 0.01.

## Data Availability

The datasets generated or analyzed during this study are available from the corresponding author on reasonable request. All code to reproduce our analyses is available at the Open Science Foundation.

## Supplementary Materials

The following supporting information can be downloaded at https://www.mdpi.com/article/10.3390/healthcare14203461/s1; Supplementary Material S1: Full survey in Dutch; Supplementary Material S2: Full survey translated from Dutch to English; Supplementary Material S3: Checklist for Reporting Results of Internet E-Surveys (CHERRIES).

## Abbreviations

The following abbreviations are used in this manuscript:

H	Hypothesis
MMRMs	Mixed Models with Repeated Measures
PDSs	Personal Data Spaces

## Appendix A

Verschillende applicaties (ook ‘apps’ genoemd) verzamelen persoonlijke gegevens over je gezondheid. Het gaat bijvoorbeeld over een bezoek aan je huisarts of een afspraak in het ziekenhuis, de resultaten van een medisch onderzoek. Maar ook het aantal stappen dat je doet per dag, informatie over je hartslag als je aan sport doet, hoe je slaapt of zelfs de voeding die je koopt. Die gegevens houden de ‘apps’ vaak bij op een plek waar je zelf niet aankan.

Hieronder geven we een beschrijving van een aanbod om met je persoonlijke gezondheidsgegevens om te gaan. Dit aanbod wordt momenteel uitgewerkt. We noemen het voorlopig “We Are”. Gelieve onderstaande beschrijving zorgvuldig te lezen vooraleer de vragen te beantwoorden.

Het gaat over het aanbieden van een veilige plek om al je persoonlijke gegevens over je gezondheid bij te houden. Deze veilige plek is een zogenaamde persoonlijke ‘datakluis’. De persoonlijke datakluis is een plek waar al jouw persoonlijke gegevens over je gezondheid worden opgeslagen. Je gegevens komen volledig onder jouw controle en beheer. Je kan dan zelf beslissen met wie je welke data deelt. Niemand kan je gegevens gebruiken als je geen toestemming hebt gegeven.

Het aanbod van “We Are” wil mensen de volledige controle bieden over het gebruiken van hun persoonlijke gezondheidsgegevens. Je hebt zelf de keuze om je persoonlijke gezondheidsgegevens te delen met zorgverleners, de overheid, onderzoeksinstellingen en bedrijven. Je kan dan je gegevens laten gebruiken om de kwaliteit van zorg te verbeteren, beleid te ontwikkelen, wetenschappelijk onderzoek te doen en om producten en diensten te verbeteren. “We Are” zal deze partijen vooraf beoordelen op een aantal ethische principes vooraleer ze de mogelijkheid te geven om via een applicatie aan jou te vragen of je je gegevens wil delen.

## Appendix B

Various applications (also known as “apps”) collect personal data about your health. This includes, for example, visits to your doctor or appointments at the hospital, the results of medical examinations, but also the number of steps you take each day, information about your heart rate when you exercise, how you sleep, or even the food you buy. The apps often store this data in a place that you cannot access yourself.

Below, we provide a description of an offer to manage your personal health data. This offer is currently being developed. We are provisionally calling it “We Are.” Please read the description below carefully before answering the questions.

It involves offering a secure place to store all your personal health data. This secure place is a so-called personal ‘data vault’. The personal data vault is a place where all your personal health data is stored. Your data is completely under your control and management. You can then decide for yourself whom you share which data with. No one can use your data without your permission.

The We Are platform aims to give people complete control over the use of their personal health data. You can choose whether to share your personal health data with healthcare providers, the government, research institutions, and companies. Your data can then be used to improve the quality of care, develop policy, conduct scientific research, and improve products and services. “We Are” will assess these parties in advance on a number of ethical principles before giving them the opportunity to ask you via an application whether you want to share your data.

## Appendix C

**Table A1:** Linear regression models predicting the two separate *We Are* evaluation scales.

	Scale 1		Scale 2	
	Model 1 B (SE)	Model 2 B (SE)	Model 1 B (SE)	Model 2 B (SE)
(Intercept)	2.497 *** (0.149)	1.590 *** (0.240)	2.206 *** (0.130)	1.663 *** (0.207)
Female (ref. = Male)	−0.067 (0.046)	0.036 (0.043)	−0.014 (0.040)	0.057 (0.037)
Age (ref. = ≤34)				
35–49 years	−0.135 + (0.069)	−0.113 + (0.064)	−0.053 (0.061)	−0.022 (0.055)
50–64 years	−0.118 (0.074)	−0.009 (0.071)	−0.056 (0.065)	0.043 (0.061)
65+ years	−0.149 (0.104)	−0.040 (0.099)	−0.080 (0.091)	0.007 (0.085)
Education (ref. = Primary educated)				
Secondary educated	0.147 * (0.069)	0.055 (0.064)	0.121 * (0.060)	0.029 (0.055)
Tertiary educated	0.285 *** (0.081)	0.135 + (0.075)	0.206 ** (0.071)	0.068 (0.065)
Main activity (ref. = private employee)				
Public employee	−0.017 (0.071)	−0.087 (0.066)	0.062 (0.062)	−0.010 (0.057)
Independent	−0.023 (0.096)	−0.048 (0.088)	0.073 (0.084)	0.032 (0.076)
Retired	0.004 (0.085)	0.059 (0.078)	0.075 (0.075)	0.113 + (0.068)
Work disabled	−0.012 (0.096)	0.022 (0.088)	0.094 (0.084)	0.105 (0.076)
Student	0.008 (0.096)	0.033 (0.088)	0.039 (0.084)	0.046 (0.076)
Other	−0.006 (0.101)	0.027 (0.093)	0.072 (0.088)	0.077 (0.080)
General self-rated health	−0.010 (0.029)	−0.044 (0.027)	0.048 + (0.026)	0.011 (0.023)
Fraud victim	0.026 (0.053)	0.011 (0.049)	0.053 (0.046)	0.051 (0.042)
Understands *We Are*		0.253 *** (0.027)		0.247 *** (0.023)
Internet knowledge		−0.027 + (0.015)		0.007 (0.013)
Privacy attitude		−0.105 ** (0.036)		−0.103 ** (0.031)
Privacy behavior		0.228 * (0.115)		−0.145 (0.100)
Trust in technology		0.161 *** (0.026)		0.130 *** (0.022)
Privacy fears technology		−0.022 (0.021)		−0.061 *** (0.018)
Technology adoption		0.079 * (0.033)		0.011 (0.028)
N	1024	1024	1024	1024
R^2^	0.034	0.197	0.024	0.209

Note: Coefficients are unstandardized, standard errors in brackets. *** *p* < 0.001, ** *p* < 0.01, * *p* < 0.05, + *p* < 0.10. Scale 1 refers to the scale with two items: attractiveness and personal relevance. Scale 2 refers to the scale with two items: credibility and trustworthiness.

## Appendix D

**Table A2:** Factor loadings for the pro-privacy attitude items.

Item	Factor 1	Factor 2
a	0.240	0.280
b	0.450	0.000
c	−0.200	0.160
d	0.610	−0.090
e	−0.110	0.700
f	0.040	0.680
g	−0.030	0.760
h	0.300	0.350
i	0.520	0.020
j	0.470	0.160
k	0.340	−0.010
l	−0.180	0.140
m	−0.040	0.240
n	0.640	−0.020
o	−0.060	−0.620
p	−0.190	−0.490
q	0.700	0.030
r	0.270	−0.100
s	0.600	0.010

Note: Factor 1 and Factor 2 columns indicate the standardized loadings from an oblique oblimin factor analysis. Items with factor loadings > 0.4 on Factor 1 were retained for the pro-privacy attitude scale.

## Appendix E

**Table A3:** Reasons for supporting *We Are* and their associations with the two separate *We Are* evaluation scales.

		Difference in *We Are*-Support (%)	
Reason for Using *We Are*	% Agree	Scale 1	Scale 2
I receive clear information about who uses my personal health data, for how long, and for what purpose	18.8 (1.2)	14.8 * (5.5)	10.7 * (4.9)
As a user of We Are, I can help decide which organizations can access the data vaults	14.8 (1.1)	25.8 *** (6.1)	19.7 *** (5.3)
I can keep all my personal health data in one place and thus maintain an overview	36.6 (1.5)	32 *** (4.4)	24.8 *** (3.9)
I don’t have to re-enter my personal health data every time it is needed for different applications	18.5 (1.2)	15 * (5.6)	14.1 ** (4.9)
I can easily access my personal health data	18.8 (1.2)	19.2 *** (5.5)	12.6 ** (4.8)
My personal health data can only be used after I give my consent	38.3 (1.5)	21.9 *** (4.4)	10.7 ** (3.9)
The privacy of my personal health data is guaranteed	30.0 (1.4)	23.6 *** (4.7)	18.2 *** (4.1)
I retain control over my personal health data	27.2 (1.4)	16.1 ** (4.9)	7.4 (4.3)
I become more aware of my own health because I have a good overview of my personal health data	7.4 (0.8)	20.8 *** (8.3)	24.5 *** (7.2)
By sharing my personal health data, I can contribute to better services and products	4.4 (0.6)	21.6 * (10.6)	17.2 (9.2)
By sharing my personal health data, I can receive better healthcare myself	14.0 (1.1)	27.9 *** (6.2)	25.1 *** (5.4)
By sharing my personal health data, I can contribute to better healthcare policy for everyone	10.3 (0.9)	30.4 *** (7.1)	34.7 *** (6.2)
I can receive tailored services in exchange for sharing my personal health data	4.7 (0.7)	−0.6 (10.3)	9.7 (9)

Note: Standard errors in brackets. *** *p* < 0.001, ** *p* < 0.01, * *p* < 0.05. Respondents were allowed to pick a maximum of three reasons. Differences in the last column result from a bivariate regression with the *We Are*-support scale and indicate the difference between those who did and did not pick this reason. Significance levels are unadjusted for multiple comparison corrections. When adjusted for multiple comparison corrections, item 10 (scale 1), and item 1 (scale 2) are not significant.

## Appendix F

**Table A4:** MMRM predicting willingness to store medical and lifestyle data in a PDS.

	Model 1 B (SE)
(Intercept)	1.794 *** (0.145)
Type (ref. = Lifestyle)	0.380 *** (0.020)
Female (ref. = Male)	−0.078 + (0.044)
Age (ref. = ≤34)	
35–49 years	−0.112 + (0.067)
50–64 years	−0.087 (0.072)
65+ years	−0.099 (0.101)
Education (ref. = Primary educated)	
Secondary educated	0.117 + (0.066)
Tertiary educated	0.218 ** (0.078)
Main activity (ref. = private employee)	
Public employee	−0.001 (0.069)
Independent	−0.132 (0.093)
Retired	0.084 (0.083)
Work disabled	0.074 (0.093)
Student	0.099 (0.093)
Other	−0.034 (0.097)
General self-rated health	0.089 (0.029)
Fraud victim	0.121 (0.051)
N (items)	15360
N (respondents)	1024
R^2^	0.082

Note: Coefficients are unstandardized, standard errors in brackets. *** *p* < 0.001, ** *p* < 0.01, + *p* < 0.10. MMRM: Mixed Model for Repeated Measures. PDS: Personal Data Space.

## Appendix G

**Table A5:** MMRM predicting willingness to share data across vignette scenarios.

	Model
(Intercept)	2.745 *** (0.195)
Item-level	
Intended purpose of data use (ref. = Public benefit commercial)	
Public benefit non-commercial	0.062 ** (0.022)
Personal benefit commercial	−0.074 *** (0.020)
Personal benefit non-commercial	0.068 *** (0.019)
Data recipient type	
Private receiver (ref. = public receiver)	−0.670 *** (0.018)
Female (ref. = Male)	0.018 (0.036)
Age (ref. = ≤34)	
35–49 years	−0.052 (0.053)
50–64 years	−0.098 + (0.059)
65+ years	−0.100 (0.082)
Education (ref. = Primary educated)	
Secondary educated	−0.035 (0.052)
Tertiary educated	−0.033 (0.062)
Main activity (ref. = private employee)	
Public employee	−0.004 (0.054)
Independent	−0.033 (0.072)
Retired	0.082 (0.065)
Work disabled	0.175 * (0.074)
Student	0.119 (0.073)
Other	0.102 (0.077)
General self-rated health	0.032 (0.023)
Fraud victim	0.091 * (0.040)
Internet knowledge	−0.000 (0.013)
Privacy attitude	−0.166 *** (0.030)
Privacy behavior	−0.462 *** (0.095)
Trust in technology	0.080 *** (0.021)
Privacy fears technology	−0.061 *** (0.017)
Technology adoption	0.114 *** (0.027)
N (items)	11823
N (respondents)	1011
R^2^	0.263

Note: Coefficients are unstandardized, standard errors in brackets. *** *p* < 0.001, ** *p* < 0.01, * *p* < 0.05, + *p* < 0.10. MMRM: Mixed Model for Repeated Measures.

## Appendix H

**Table A6:** Classification of the vignette scenarios by intended purpose and recipient type.

Item number	Item	Receiver	Personal/Public	(Non-)Commercial
1	Sharing data from your data vault with a commercial company (an “app”) to receive personalized health advice	Private	Personal	Non-commercial
2	Sharing data about your lifestyle (exercise, nutrition, sleep patterns, etc.) with your family doctor to receive more tailored care	Public	Personal	Non-commercial
3	Sharing data about your hospital admissions with pharmaceutical companies for the development of new medicines	Private	Public	Non-commercial
4	Sharing data about your health with a research institute for scientific research into diseases and treatments	Public	Public	Non-commercial
5	Sharing data about your general health status with health insurance funds/mutual insurance companies to determine a service tailored to your situation	Public	Personal	Non-commercial
6	Sharing data about your lifestyle (exercise, diet, sleep patterns, etc.) with the government to determine health policy (prevention, screening, planning)	Public	Public	Non-commercial
7	Sharing data about your blood tests with insurance companies to adjust your premium to your health status	Private	Personal	Non-commercial
8	Sharing data about your general health status with health insurance funds/mutual insurance companies to determine reimbursements for medical expenses tailored to your situation	Public	Personal	Commercial
9	Sharing data about your purchasing behavior regarding medicines with a pharmaceutical company to support the marketing of their products	Private	Public	Commercial
10	Sharing data in your health vault with an emergency service so they are informed about your health when you need medical intervention	Public	Personal	Non-commercial
11	Sharing data about your experiences during a hospital admission with the hospital to improve care	Public	Public	Non-commercial
12	Sharing data from your data vault with a commercial company (an “app”) to get a discount on your next purchase	Private	Personal	Commercial

Note: Personal benefit refers to uses that provide a direct benefit to the individual, whereas public benefit refers to uses whose purpose extends beyond a direct individual benefit. Commerciality was classified separately. For vignette 9, concerning pharmaceutical marketing, the classification as public benefit indicates that the described purpose does not provide a direct personal benefit to the individual. Unlike other public benefit scenarios, however, a broader societal benefit is not explicitly stated in the vignette wording. This scenario should therefore be considered a less direct operationalization of public benefit.
