# Supplementary material for: Public Perceptions of Personal Data Spaces for Citizen-Centered Health Data Governance in Belgium: Cross-Sectional Survey
Source: Healthcare (Basel). 2026 Oct 15;14(20):3461. doi: 10.3390/healthcare14203461 (PMC13704102; doi:10.3390/healthcare14203461)
Supplement: Supplementary file 1 [file healthcare-14-03461-s001.zip › Supplementary Material S1.pdf]

Supplementary Material S1: Full survey in Dutch

General evaluation of PDS

Hoe aantrekkelijk vind je dit aanbod van “We are”?

- Zeer aantrekkelijk
- Aantrekkelijk
- Enigszins aantrekkelijk
- Niet erg aantrekkelijk
- Zeer onaantrekkelijk

In hoeverre vind je dit aanbod van “We Are” relevant voor jezelf?

- Helemaal niet relevant
- Eerder niet relevant
- Eerder relevant
- Heel relevant

In hoeverre vind je het aanbod van “We Are” geloofwaardig?

- Helemaal niet geloofwaardig
- Eerder niet geloofwaardig
- Geloofwaardig
- Heel geloofwaardig

In hoeverre vertrouw je het aanbod van “We Are”?

- Ik vertrouw dit helemaal niet
- Ik vertrouw dit eerder niet
- Ik vertrouw dit eerder wel
- Ik vertrouw dit helemaal

Reasons for supporting PDS

Hieronder zie je een lijst met verschillende redenen om *We Are* te gebruiken. Kan je de redenen aanduiden die jou het meeste overtuigen om *We Are* te gebruiken? Je kan maximum 3 redenen aanvinken.

- Ik krijg duidelijke informatie over wie allemaal mijn persoonlijke gezondheidsgegevens gebruikt, hoelang en waarvoor
- Als gebruiker van *We Are* kan ik mee beslissen over welke organisaties toegang kunnen krijgen tot de datakluizen
- Al mijn persoonlijke gezondheidsgegevens op één plaats kunnen bijhouden en zo een overzicht behouden
- Mijn persoonlijke gezondheidsgegevens niet steeds opnieuw in moeten geven als die nodig zijn voor verschillende toepassingen
- Op een gemakkelijke manier toegang kunnen krijgen tot mijn persoonlijke gezondheidsgegevens
- Mijn persoonlijke gezondheidsgegevens kunnen enkel gebruikt worden nadat ik toestemming geef
- De privacy van mijn persoonlijke gezondheidsgegevens wordt gegarandeerd
- Ik houd controle over mijn persoonlijke gezondheidsgegevens
- Ik word me meer bewust over mijn eigen gezondheid doordat ik een goed zicht heb op mijn persoonlijke gezondheidsgegevens
- Door mijn persoonlijke gezondheidsgegevens te delen kan ik een bijdrage leveren aan betere diensten en producten
- Door mijn persoonlijke gezondheidsgegevens te delen kan ik zelf een betere zorgverlening krijgen
- Door mijn persoonlijke gezondheidsgegevens te delen kan ik een bijdrage leveren aan een beter beleid voor de gezondheid van iedereen
- Ik kan diensten krijgen op mijn maat in ruil voor het delen van mijn persoonlijke gezondheidsgegevens
- Geen van de bovenstaande

Participation preferences in PDS

In welke mate ben je het eens of oneens met de onderstaande uitspraken?

Ik deel alleen persoonlijke gegevens over mijn gezondheid als ik weet waarvoor ze gebruikt worden.

- Helemaal oneens
- Oneens

- Noch eens, noch oneens
- Eens
- Helemaal eens

Vooraleer ik mijn persoonlijke gegevens deel, wil ik weten waar ze voor gebruikt worden.

- Helemaal oneens
- Oneens
- Noch eens, noch oneens
- Eens
- Helemaal eens

Ik vind het belangrijk om zelf controle te hebben over met wie precies ik welke gegevens deel.

- Helemaal oneens
- Oneens
- Noch eens, noch oneens
- Eens
- Helemaal eens

Types of data stored in PDS

Kan je hieronder aangeven in welke mate je onderstaande persoonlijke gegevens over je gezondheid in de datakluis "We Are" zou willen bewaren? Helemaal niet – eerder niet – eerder wel – helemaal wel.

a) Gegevens over de voeding die je aankoopt
b) Gegevens over bloedafnames, zoals je bloedgroep en testresultaten
c) Gegevens over je bezoeken aan de huisarts en andere zorgverleners
d) Resultaten van medische onderzoeken in het ziekenhuis
e) Vaccinatiegegevens (welke vaccinaties en wanneer, enz.)
f) Gegevens over de medicatie die je aankoopt bij de apotheek
g) Gegevens over ziekenhuisopnames
h) Gegevens over hoe vaak je sport en beweegt

i) Gegevens die worden uitgewisseld tijdens consultaties in het ziekenhuis

j) Gegevens over de dranken die je aankoopt (alcohol, zuivel, water, frisdrank, enz.)

k) Gegevens over je slaappatroon

l) Gegevens over je DNA

m) Gegevens over alcoholtesten en speekseltesten

n) Gegevens over bezoeken aan de arbeidsgeneeskundige dienst

o) Gegevens over consultaties bij een psycholoog of psychiater

Intended purpose of data use in PDS

Stel dat je de datakluis "We Are" kan gebruiken. Hieronder zie je enkele concrete situaties. Kan je aanduiden in hoeverre je bereid bent om je persoonlijke gezondheidsdata te delen in de voorgelegde situaties? Helemaal niet bereid tot delen – eerder niet bereid tot delen – eerder bereid tot delen – helemaal bereid tot delen – ik weet het niet.

a) Gegevens uit je datakluis delen met een commercieel bedrijf (een 'app') om gepersonaliseerd gezondheidsadvies te krijgen

b) Gegevens over je levensstijl (zoals beweging, voeding, slaappatroon, …) delen met je huisarts om meer zorg op jouw maat te krijgen

c) Gegevens over je opnames in het ziekenhuis delen met farmaceutische bedrijven voor de ontwikkeling van nieuwe geneesmiddelen

d) Gegevens over je gezondheid delen met een onderzoeksinstelling voor wetenschappelijk onderzoek naar ziekten en behandelingen

e) Gegevens over je algemene gezondheidstoestand delen met ziekenfondsen/mutualiteiten om een dienstverlening aangepast aan jouw situatie te bepalen

f) Gegevens over je levensstijl (zoals beweging, voeding, slaappatroon, …) met de overheid delen om het beleid inzake gezondheid te bepalen (preventie, screening, planning …)

g) Gegevens over je bloedafnames delen met verzekeringsmaatschappijen om het bedrag van een premie aan te passen aan jouw gezondheidstoestand

h) Gegevens over je algemene gezondheidstoestand delen met ziekenfondsen/mutualiteiten om terugbetalingen van ziektekosten op maat van jouw situatie te bepalen

i) Gegevens over je aankoopgedrag van medicijnen delen met een farmaceutische firma om de marketing van hun producten te ondersteunen

j) Data in je gezondheidskluis delen met een spoeddienst zodat ze geïnformeerd zijn over je gezondheid wanneer je een interventie nodig hebt

k) Gegevens over je ervaringen bij een opname in het ziekenhuis delen met het ziekenhuis om de zorg te verbeteren

l) Gegevens uit je datakluis delen met een commercieel bedrijf (een 'app') om een korting op je volgende aankoop te krijgen

Gender

Wat is je geslacht?

- Man
- Vrouw
- X

Age

Wat is je leeftijd?

Educational level

Wat is je hoogst behaalde diploma?

- Geen diploma
- Lager onderwijs
- Lager secundair onderwijs
- Hoger secundair onderwijs
- Hoger onderwijs (hogeschool of universiteit)

Main activity

Hoe kan je alledaagse activiteit het best omschreven worden vooraleer je op pensioen ging?

- Arbeider in de privésector (profit of non-profit)
- Bediende in de privésector (profit of non-profit)

- Statutair ambtenaar in de openbare sector of het onderwijs
- Contractuele werknemer in de openbare sector of het onderwijs
- Zelfstandige (vrij beroep, handelaar, freelancer, bestuurder ...) zonder personeel
- Zelfstandige (ondernemer, bedrijfsleider, ...) met personeel
- Helper – medewerkend familielid zonder vergoeding
- Huisvrouw/man, je verzorgt dus het eigen huishouden
- Werkzoekend/werkloos
- Permanent of langdurig arbeidsongeschikt (invaliditeit, ziekte, ...)
- In volledige loopbaanonderbreking (of tijdskrediet) langer dan 3 maanden
- Andere situatie zonder betrekking

General self-rated health

Hoe is je gezondheidstoestand in het algemeen?

- Zeer slecht
- Slecht
- Gaat wel (redelijk)
- Goed
- Zeer goed

Fraud victimhood

Ben jij of iemand uit je huishouden ooit het slachtoffer geweest van oplichting of fraude?

- Ja, voor een bedrag minder dan €100
- Ja, voor een bedrag tussen €100 en €500
- Ja, voor een bedrag hoger dan €500
- Nee
- Zeg ik liever niet

Internet knowledge

Hoe zou je zelf je vertrouwdheid met het internet (zowel websites of apps) beoordelen?

Je kan antwoorden op een schaal van 0 tot 10 waarbij 0 'Helemaal niet vertrouwd met het internet' betekent en 10 'heel vertrouwd met het internet' betekent. De tussenliggende scores dienen om je antwoord te nuanceren.

0	1	2	3	4	5	6	7	8	9	10
❍	❍	❍	❍	❍	❍	❍	❍	❍	❍	❍

Pro-privacy attitude

In welke mate ben je het eens of oneens met de onderstaande uitspraken?

	Helemaal oneens	Oneens	Noch eens, noch oneens	Eens	Helemaal eens
a) Ik ben bezorgd dat mijn persoonlijke online gegevens onveilig worden bewaard	❍	❍	❍	❍	❍
b) Ik aanvaard gemakkelijk alle cookies als ik een website bezoek	❍	❍	❍	❍	❍
c) Ik kan misbruik van mijn online gegevens door bedrijven met slechte bedoelingen onmogelijk tegenhouden	❍	❍	❍	❍	❍
d) Ik denk goed na voor ik een applicatie toegang geef tot mijn locatie, microfoon, contactlijst, camera, ...	❍	❍	❍	❍	❍
e) Ik vind het gevaarlijk om persoonlijke gegevens over mijn gezondheid te delen met bedrijven	❍	❍	❍	❍	❍

f) Ik vind het gevaarlijk om persoonlijke gegevens over mijn gezondheid te delen met zorgverleners	❍	❍	❍	❍	❍
g) Ik vind het gevaarlijk om persoonlijke gegevens over mijn gezondheid te delen met de overheid	❍	❍	❍	❍	❍
h) Ik vind het gevaarlijk om persoonlijke gegevens over mijn gezondheid te delen voor wetenschappelijk onderzoek	❍	❍	❍	❍	❍
i) Ik deel gemakkelijk gegevens online in ruil voor gratis diensten	❍	❍	❍	❍	❍
j) Ik lig er niet wakker van hoe online gegevens over mij worden gebruikt	❍	❍	❍	❍	❍
k) Ik deel alleen persoonlijke gegevens over mijn gezondheid als ik weet waarvoor ze gebruikt worden	❍	❍	❍	❍	❍
l) Ik heb geen zicht op wat met mijn persoonlijke gegevens op het internet precies gebeurt	❍	❍	❍	❍	❍
m) Ik heb geregeld hulp nodig van anderen bij het installeren van apps	❍	❍	❍	❍	❍
n) Vooraleer ik mijn persoonlijke gegevens deel, wil ik weten waar ze voor gebruikt worden	❍	❍	❍	❍	❍
o) Ik ben bereid om mijn gegevens over mijn gezondheid te delen als dat de samenleving ten goede komt	❍	❍	❍	❍	❍

p) Ik ben bereid om mijn gegevens over mijn gezondheid te delen ook als ik er zelf geen rechtstreeks voordeel bij haal					
q) Ik deel gemakkelijk mijn gegevens online om tijd te besparen	❍	❍	❍	❍	❍
r) Ik deel alleen mijn persoonlijke gegevens als het duidelijk is welke voordelen er voor mij aan verbonden zijn	❍	❍	❍	❍	❍
s) Ik vind het belangrijk om zelf controle te hebben over met wie precies ik welke gegevens deel	❍	❍	❍	❍	❍

Pro-privacy behavior

De volgende vraag gaat over bepaalde zaken die je kan doen om je online privacy te beschermen. Kan je aanduiden welke van deze zaken je bewust hebt gedaan in de voorbije drie maanden?

Je kan meerdere antwoorden aanvinken

- Geen toegang tot sommige gegevens geven
- Bepaalde apps bewust niet downloaden
- Beveiligings- of anti-trackingsoftware gebruiken
- Mijn wachtwoorden wijzigen
- De privacy instellingen van sociale media zoals bijvoorbeeld facebook of Instagram aanpassen
- Cookies verwijderen
- De privacy instellingen (via 'instellingen') op mijn smartphone aanpassen
- Zo weinig mogelijk cookies accepteren
- Applicaties of accounts verwijderen
- Geen publieke wifi-netwerken gebruiken
- In 'incognito'-modus surfen
- Valse informatie bij een registratie gebruiken (zoals leeftijd, postcode, geslacht,…)

- Bepaalde producten bewust niet meer online kopen
- Mijn locatie uitschakelen op mijn smartphone
- Mijn bluetooth uitzetten op mijn smartphone
- Een password-tool/wachtwoordmanager gebruiken
- Een VPN gebruiken
- Ik deed geen van bovenstaande zaken de voorbije drie maanden

Trust in technology

In welke mate heb je vertrouwen dat de technologische ontwikkelingen die nu volop in een stroomversnelling zitten (zoals zelfrijdende wagens, digitalisering, artificiële intelligentie, automatisatie) een meerwaarde voor jou zullen betekenen in de toekomst?

Geef een score van 1 tot 5 (1 = Heel weinig vertrouwen - 5 = Heel veel vertrouwen).

Heel weinig				Heel veel
1	2	3	4	5
❍	❍	❍	❍	❍

Privacy fears due to technology

In welke mate heb je schrik dat de technologische ontwikkelingen zullen leiden tot een samenleving waar privacy niet of nauwelijks bestaat?

Geef een score van 1 tot 5 (1 = Heel weinig schrik - 5 = Heel veel schrik).

Heel weinig				Heel veel
1	2	3	4	5
❍	❍	❍	❍	❍

Technological adoption

In welke mate ga je akkoord met de volgende uitspraken over nieuwe technologieën?

Source: Based on Domain Specific Innovativeness (DSI) scale developed by Goldsmith & Hofacker (1991) and previously tested and used, among others, in eGovernment and ICT adoption related research by University of Antwerp (Belgium) in collaboration with Indiville.

	Helemaal niet akkoord	Niet akkoord	Neutraal	Akkoord	Helemaal akkoord	Weet niet
Over het algemeen volg ik de laatste nieuwe trends	❍	❍	❍	❍	❍	❍
Ik gebruik onmiddellijk nieuwe internettoepassingen, anders blijf ik achter	❍	❍	❍	❍	❍	❍
Wanneer ik verneem dat ze beschikbaar zijn, dan ben ik voldoende geïnteresseerd om nieuwe internettoepassingen te gebruiken	❍	❍	❍	❍	❍	❍
In vergelijking met mijn vrienden gebruik ik niet veel internettoepassingen	❍	❍	❍	❍	❍	❍
In vergelijking met mijn vrienden weet ik niet veel over internettoepassingen	❍	❍	❍	❍	❍	❍
Over het algemeen ben ik de eerste in mijn kennissenkring die op de hoogte is over de	❍	❍	❍	❍	❍	❍

meest recente internettoepassingen						
Ik gebruik niet graag nieuwe internetapplicaties voor andere mensen dat hebben gedaan	❍	❍	❍	❍	❍	❍
Over het algemeen ben ik bij de eersten in mijn kennissenkring om nieuwe internettoepassingen te gebruiken.	❍	❍	❍	❍	❍	❍
