# Supplementary material for: Public Perceptions of Personal Data Spaces for Citizen-Centered Health Data Governance in Belgium: Cross-Sectional Survey
Source: Healthcare (Basel). 2026 Oct 15;14(20):3461. doi: 10.3390/healthcare14203461 (PMC13704102; doi:10.3390/healthcare14203461)
Supplement: Supplementary file 1 [file healthcare-14-03461-s001.zip › Supplementary Material S2.pdf]

Supplementary Material S2: Full survey translated from Dutch to English

General evaluation of PDSs

How appealing do you find this offer from We Are?

- Very appealing
- Appealing
- Somewhat appealing
- Not very appealing
- Very unappealing

To what extent do you find this offer from We Are relevant to yourself?

- Not at all relevant
- Rather irrelevant
- Rather relevant
- Very relevant

To what extent do you find the offer from We Are credible?

- Not at all credible
- Somewhat credible
- Credible
- Very credible

To what extent do you trust the offer from We Are?

- I do not trust it at all
- I do not particularly trust it
- I trust it somewhat
- I fully trust this

Reasons for supporting PDSs

Below is a list of different reasons to use *We Are*. Can you indicate the reasons that most convince you to use *We Are*? You can select a maximum of 3 reasons.

- I receive clear information about who uses my personal health data, for how long, and for what purpose
- As a user of We Are, I can help decide which organizations can access the data vaults
- I can keep all my personal health data in one place and thus maintain an overview
- I don't have to re-enter my personal health data every time it is needed for different applications
- I can easily access my personal health data
- My personal health data can only be used after I give my consent
- The privacy of my personal health data is guaranteed
- I retain control over my personal health data
- I become more aware of my own health because I have a good overview of my personal health data
- By sharing my personal health data, I can contribute to better services and products
- By sharing my personal health data, I can receive better healthcare myself
- By sharing my personal health data, I can contribute to better healthcare policy for everyone
- I can receive tailored services in exchange for sharing my personal health data
- None of the above

Participation preferences in PDSs

To what extent do you agree or disagree with the statements below?

I only share personal health data if I know what they will be used for.

- Strongly disagree
- Disagree
- Neither agree nor disagree
- Agree
- Strongly agree

Before I share my personal data, I want to know what they will be used for.

- Strongly disagree

- Disagree
- Neither agree nor disagree
- Agree
- Strongly agree

I find it important to have control over whom I share which data with.

- Strongly disagree
- Disagree
- Neither agree nor disagree
- Agree
- Strongly agree

Types of data stored in PDSs

Please indicate below to what extent you would like to store the following personal health data in the *We Are* data vault. Definitely not—rather not—somewhat—definitely yes.

a) Data about blood tests (e.g., blood type and results)

b) Data about visits to your GP and other healthcare providers

c) Results of medical examinations at the hospital

d) Vaccination data (which vaccines and when)

e) Data about the medication you purchase at the pharmacy

f) Data about hospital admissions

g) Data exchanged during consultations at the hospital

h) Data about your DNA

i) Data about alcohol tests and saliva tests

j) Data about visits to an occupational health service

k) Data about consultations with a psychologist or psychiatrist

l) Data about the food you buy

m) Data about how often you exercise and play sports

n) Data about the beverages you purchase (alcohol, dairy, water, soft drinks, etc.)

o) Data about your sleeping habits

Intended purpose of data use in PDSs

Suppose you can use the *We Are* data vault. Below are some specific situations. Can you indicate to what extent you are willing to share your personal health data in the situations presented? Not at all willing to share—rather unwilling to share—rather willing to share—completely willing to share—I don't know.

a) Sharing data from your data vault with a commercial company (an "app") to receive personalized health advice

b) Sharing data about your lifestyle (exercise, nutrition, sleep patterns, etc.) with your family doctor to receive more tailored care

c) Sharing data about your hospital admissions with pharmaceutical companies for the development of new medicines

d) Sharing data about your health with a research institute for scientific research into diseases and treatments

e) Sharing data about your general health status with health insurance funds/mutual insurance companies to determine a service tailored to your situation

f) Sharing data about your lifestyle (exercise, diet, sleep patterns, etc.) with the government to determine health policy (prevention, screening, planning)

g) Sharing data about your blood tests with insurance companies to adjust your premium to your health status

h) Sharing data about your general health status with health insurance funds/mutual insurance companies to determine reimbursements for medical expenses tailored to your situation

i) Sharing data about your purchasing behavior regarding medicines with a pharmaceutical company to support the marketing of their products

j) Sharing data in your health vault with an emergency service so they are informed about your health when you need medical intervention

k) Sharing data about your experiences during a hospital admission with the hospital to improve care

l) Sharing data from your data vault with a commercial company (an "app") to get a discount on your next purchase

Gender

What is your gender?

- o Male
- o Female
- o X

Age

What is your age?

Educational level

What is your highest level of education?

- o No diploma
- o Primary education
- o Lower secondary education
- o Upper secondary education
- o Higher education (college or university)

Main activity

How would you best describe your daily activities before you retired?

- o Worker in the private sector (profit or non-profit)
- o Employee in the private sector (profit or non-profit)
- o Statutory civil servant in the public sector or education
- o Contractual employee in the public sector or education
- o Self-employed (liberal profession, trader, freelancer, director, etc.) without staff
- o Self-employed (entrepreneur, business manager, etc.) with staff
- o Helper—assisting family member without remuneration
- o Housewife/househusband, i.e., you take care of your own household
- o Job seeker/unemployed
- o Permanently or long-term incapacitated for work (disability, illness, etc.)
- o On full career break (or time credit) for more than 3 months

- Other situation without employment

General self-rated health

How would you describe your overall health?

- Very poor
- Poor
- Fair
- Good
- Very good

Fraud victimhood

Have you or anyone in your household ever been the victim of fraud or scams?

- Yes, for an amount less than €100
- Yes, for an amount between €100 and €500
- Yes, for an amount greater than €500
- No
- I'd rather not say

Internet knowledge

How would you rate your familiarity with the internet (both websites and apps)?

You can answer on a scale of 0 to 10, where 0 means 'Not at all familiar with the internet' and 10 means 'Very familiar with the internet'. The scores in between are used to nuance your answer.

0	1	2	3	4	5	6	7	8	9	10
❍	❍	❍	❍	❍	❍	❍	❍	❍	❍	❍

Pro-privacy attitude

To what extent do you agree or disagree with the statements below?

	Strongly disagree	Disagree	Neither agree nor disagree	Agree	Strongly agree

a) I am concerned that my personal online data is not stored securely	❍	❍	❍	❍	❍
b) I readily accept all cookies when I visit a website	❍	❍	❍	❍	❍
c) I cannot prevent companies with malicious intent from misusing my online data	❍	❍	❍	❍	❍
d) I think carefully before giving an application access to my location, microphone, contact list, camera, etc.	❍	❍	❍	❍	❍
e) I think it is dangerous to share personal data about my health with companies	❍	❍	❍	❍	❍
f) I think it is dangerous to share personal data about my health with healthcare providers	❍	❍	❍	❍	❍
g) I think it is dangerous to share personal data about my health with the government	❍	❍	❍	❍	❍
h) I think it is dangerous to share personal data about my health for scientific research	❍	❍	❍	❍	❍
i) I readily share data online in exchange for free services	❍	❍	❍	❍	❍
j) I don't lose sleep over how my online data is used	❍	❍	❍	❍	❍

k) I only share personal data about my health if I know what it will be used for	❍	❍	❍	❍	❍
l) I have no idea what exactly happens to my personal data on the internet	❍	❍	❍	❍	❍
m) I regularly need help from others to install apps	❍	❍	❍	❍	❍
n) Before I share my personal data, I want to know what it will be used for	❍	❍	❍	❍	❍
o) I am willing to share my health data if it benefits society	❍	❍	❍	❍	❍
p) I am willing to share my health data even if I do not directly benefit from it myself	❍	❍	❍	❍	❍
q) I readily share my data online to save time	❍	❍	❍	❍	❍
r) I only share my personal data if the benefits to me are clear	❍	❍	❍	❍	❍
s) I think it is important to have control over exactly who I share which data with	❍	❍	❍	❍	❍

Pro-privacy behavior

The next question concerns certain things you can do to protect your online privacy. Can you indicate which of these things you have consciously done in the past three months?

You can select multiple answers.

- o Not grant access to certain data
- o Deliberately refrain from downloading certain apps
- o Use security or anti-tracking software

- Change my passwords
- Adjust the privacy settings of social media such as Facebook or Instagram
- Delete cookies
- Adjust the privacy settings (via ‘settings’) on my smartphone
- Accept as few cookies as possible
- Delete applications or accounts
- Not use public Wi-Fi networks
- Browse in incognito mode
- Use false information when registering (such as age, postal code, gender, etc.)
- Deliberately stop buying certain products online
- Turn off location services on my smartphone
- Turn off Bluetooth on my smartphone
- Use a password tool/password manager
- Use a Virtual Private Network (VPN)
- I have not done any of the above in the past three months

Trust in technology

To what extent are you confident that the technological developments that are currently gaining momentum (such as self-driving cars, digitization, artificial intelligence, automation) will add value for you in the future?

Give a score from 1 to 5 (1 = Very little confidence - 5 = Very confident).

Very little confidence				Very confident
1	2	3	4	5
❍	❍	❍	❍	❍

Privacy fears due to technology

To what extent are you concerned that technological developments will lead to a society where privacy is non-existent or virtually non-existent?

Give a score from 1 to 5 (1 = Very little concern - 5 = Very great concern).

Very little concern				Very great concern
1	2	3	4	5
❍	❍	❍	❍	❍

Technological adoption

To what extent do you agree with the following statements about new technologies?

Source: Based on the Domain Specific Innovativeness (DSI) scale developed by Goldsmith & Hofacker (1991) and previously tested and used, among others, in eGovernment and ICT adoption-related research by the University of Antwerp (Belgium) in collaboration with Indiville.

	Strongly disagree	Disagree	Neutral	Agree	Strongly agree	Don’t know
In general, I follow the latest trends.	❍	❍	❍	❍	❍	❍
I immediately use new internet applications, otherwise I fall behind.	❍	❍	❍	❍	❍	❍
When I hear that they are available, I am interested enough to use new internet applications.	❍	❍	❍	❍	❍	❍
Compared to my friends, I don't use internet applications very much.	❍	❍	❍	❍	❍	❍

Compared to my friends, I don't know much about internet applications.	❍	❍	❍	❍	❍	❍
In general, I am the first among my friends to know about the latest internet applications.	❍	❍	❍	❍	❍	❍
I don't like to use new internet applications that other people have used.	❍	❍	❍	❍	❍	❍
In general, I am among the first among my friends to use new internet applications.	❍	❍	❍	❍	❍	❍
