# Supplementary material for: Public Perceptions of Personal Data Spaces for Citizen-Centered Health Data Governance in Belgium: Cross-Sectional Survey
Source: Healthcare (Basel). 2026 Oct 15;14(20):3461. doi: 10.3390/healthcare14203461 (PMC13704102; doi:10.3390/healthcare14203461)
Supplement: Supplementary file 1 [file healthcare-14-03461-s001.zip › Supplementary Material S3.pdf]

Supplementary Material S3: Checklist for Reporting Results of Internet E-Surveys (CHERRIES).

	**Checklist for Reporting Results of Internet E-Surveys (CHERRIES)**	
Item Category	***Checklist Item***	***Explanation***
**Design**		
	Describe survey design	Internet-based survey using an opt-in sample of adults aged 18-79 in Flanders, Belgium, recruited via an existing online panel and matched to demographic quotas for gender, age, education, province, and urbanization level.
**IRB (Institutional Review Board) approval and informed consent process**		
	IRB approval	Ethical approval was obtained from the Ethics Committee of University Hospital of Antwerp (UZA)/UAntwerp (BUN300201940638).
	Informed consent	Participants received information about the survey length in the recruitment email. Before starting the survey, they were informed that participation was voluntary, that responses would be treated anonymously and confidentially, and that data would

	Checklist for Reporting Results of Internet E-Surveys (CHERRIES)	
Item Category	*Checklist Item*	*Explanation*
		be analyzed only at an aggregated level. Potentially sensitive questions were explained as necessary for the research.
	Data protection	Responses were anonymized and stored securely by the survey administrator. No IP addresses were collected. Data were transferred in anonymized form to the research institution.
**Development and pre-testing**		
	Development and testing	The survey was developed based on the literature and in collaboration with partners from the *We Are* initiative, including representatives from research institutions, healthcare organizations, general practitioners, and patient organizations (including patient experience experts). It was pretested with 10 participants for usability, clarity, and technical functionality.
**Recruitment process and description of the sample having access to the questionnaire**		

	Checklist for Reporting Results of Internet E-Surveys (CHERRIES)	
Item Category	*Checklist Item*	*Explanation*
	Open survey versus closed survey	Closed survey. Only invited panel members could participate.
	Contact mode	Participants were invited via email through the panel provider.
	Advertising the survey	No public advertising; invitations were sent directly to panel members without disclosure of the survey topic.
**Survey administration**		
	Web/E-mail	Web-based survey administered online via a panel platform. Responses were automatically captured.
	Context	The survey was conducted within an online research panel environment, consisting of individuals who have registered to participate in surveys on societal topics.
	Mandatory/voluntary	Participation was voluntary.
	Incentives	Participants received points through the panel’s incentive system, which could be exchanged for monetary rewards or charity donations.
	Time/Date	Data were collected in January 2024.

	**Checklist for Reporting Results of Internet E-Surveys (CHERRIES)**	
Item Category	*Checklist Item*	*Explanation*
	Randomization of items or questionnaires	Item order within question batteries was randomized.
	Adaptive questioning	Adaptive questioning was used to display only relevant questions based on prior responses.
	Number of Items	The survey consisted of multiple thematic blocks; questions were presented individually (one per screen on mobile).
	Number of screens (pages)	The survey comprised 30 pages with questions (32 pages in total including final screen).
	Completeness check	All items required a response; participants could select options such as “prefer not to answer” where applicable.
	Review step	Participants could review and change their answers before submission through the back button.
**Response rates**		
	Unique site visitor	Not applicable due to panel-based recruitment.
	View rate (ratio of unique survey visitors/unique site visitors)	Not available due to panel-based recruitment.

	**Checklist for Reporting Results of Internet E-Surveys (CHERRIES)**	
Item Category	*Checklist Item*	*Explanation*
	Participation rate (ratio of unique visitors who agreed to participate/unique first survey page visitors)	Not available. The number of panel members invited to participate was not available to the researchers.
	Completion rate (ratio of users who finished the survey/users who agreed to participate)	Not available. Incomplete responses were excluded before delivery of the final dataset, and the number of incomplete responses was not available to the researchers.
**Preventing multiple entries from the same individual**		
	Cookies used	Cookies were used to allow respondents to resume the survey where they left off.
	IP check	No IP addresses were collected.
	Log file analysis	No additional log file analysis was performed beyond standard data screening procedures.
	Registration	Participants accessed the survey via personalized invitation links through a registered panel account and could complete the survey only once.
**Analysis**		

	**Checklist for Reporting Results of Internet E-Surveys (CHERRIES)**	
Item Category	***Checklist Item***	***Explanation***
	Handling of incomplete questionnaires	Only completed questionnaires were included in the analysis.
	Questionnaires submitted with an atypical timestamp	Response durations were assessed together with variation across scale items, rather than using a fixed exclusion threshold based solely on completion time. Implausibly fast or otherwise questionable responses were reviewed on a case-by-case basis and excluded where appropriate. This review was conducted by Indiville before delivery of the final dataset, and the exact number of responses excluded during this process was not available to the researchers.
	Statistical correction	No statistical weighting or correction was applied; demographic quotas were used for gender, age, education, province, and urbanization level.
